# The non-unitary nature of information preference

**DOI:** 10.3758/s13423-022-02243-5

**Published:** 2023-04-19

**Authors:** Shi Xian Liew, Jake R. Embrey, Ben R. Newell

**Affiliations:** School of Psychology, UNSW, Sydney, NSW Australia

**Keywords:** Information-seeking, Information preference, Individual differences, Trait-measurement, Non-instrumentality

## Abstract

Factors affecting information-seeking behaviour can be task-endogenous (e.g., probability of winning a gamble), or task-exogenous (e.g., personality trait measures). Various task-endogenous factors affecting non-instrumental information-seeking behaviour have been identified, but it is unclear how task-exogenous factors affect such behaviour, and if they interact with task-endogenous factors. In an online information seeking experiment (*N* = 279), we focus on the role that outcome probability, as a task-endogenous factor, has on information preferences. We find reliable preference for advance information on highly probable gains and low preference for highly probable losses. Comparisons with individual trait measures of information preference (e.g., intolerance of uncertainty scale, obsessive-compulsive inventory, information preferences scale) reveal minimal association between these task-exogenous factors with choice task performance. We also find minimal interaction between outcome probability and individual trait measures. Despite the choice task and trait measures purportedly tapping the same (or similar) construct, the absence of clear relationships ultimately suggests a multi-dimensional nature of information preference.

**Supplementary Information:**

The online version contains supplementary material available at 10.3758/s13423-022-02243-5.

## Introduction

Information preference may be driven by any multitude of factors, from changes in an ongoing situation to the personal tendencies of an agent. For instance, we may expect investors to be more aggressive in monitoring share prices when there is greater stock market volatility. Similarly, we can expect monitoring behaviour to vary depending on the investor’s personality traits—anxious investors may check their portfolio more frequently. It would also be unsurprising if these factors interacted—more anxious investors may be especially keen to watch the market during periods of volatility and vice versa. In this example, we have assumed that these different task-endogenous (market volatility) and task-exogenous (trait anxiety) factors relate to a single concept known as information preference, but to what extent do these different factors actually index the same construct?

The information preference literature typically distinguishes non-instrumental from instrumental information, where the former can be used to guide future action and the latter cannot. With instrumental information, we may expect a relationship between task-exogenous measures and a relevant task. Using the earlier example, more anxious investors may more actively seek information that can guide their future actions (e.g., to sell or buy more stocks). With non-instrumental information, however, the relationship between task-exogenous factors and the task itself is less clear, simply because the information people can obtain cannot be used to change their situation. Empirically, while some non-instrumental information-seeking studies have measured the relationship between various factors and task performance, any relationships found tend to be relatively small, with no clear link between task-exogenous and task-endogenous factors (e.g., Bennett, Sutcliffe, Tan, Smillie, & Bode, 2021; Jach, DeYoung, & Smillie, 2021), but for some evidence of a link between anxiety and information seeking in particular environments see Charpentier et al., 2022).

Several authors have recently begun to attempt to understand the structure of information preference. Sharot and Sunstein (2020) developed a conceptual framework outlining three motivations to seek information. Specifically, they argued information is sought for its value in supplying instrumental utility (i.e., information that guides future action), hedonic utility (e.g., information inducing positive affect), and cognitive utility (e.g., information for understanding reality). These motivations are all sourced from the nature of the information itself—-that is, they are task-endogenous factors of information-seeking. While Sharot and Sunstein (2020) have described how we might be able to understand different task-endogenous factors of information preference, their framework does not explicitly identify how task-exogenous factors can contribute to the decision process (but for recent progress in this direction see Kelly et al., 2021).

Conversely, Jach et al., (2021) adopted a different approach by proposing two pathways for information preference that are primarily driven by task-exogenous factors. The first is described by traits relating to the exploration of unknowns (e.g., curiosity and openness), while the second involves traits relevant to safety-seeking (e.g., uncertainty intolerance and negative emotionality). Across two non-instrumental information studies involving trivia games and coin flips, Jach et al., (2021) found that openness and curiosity traits were related to information preference on the former, while uncertainty intolerance was only partially related to the latter. Despite a wide array of other task-exogenous measures, no other substantial associations were noted.

**Fig. 1:**
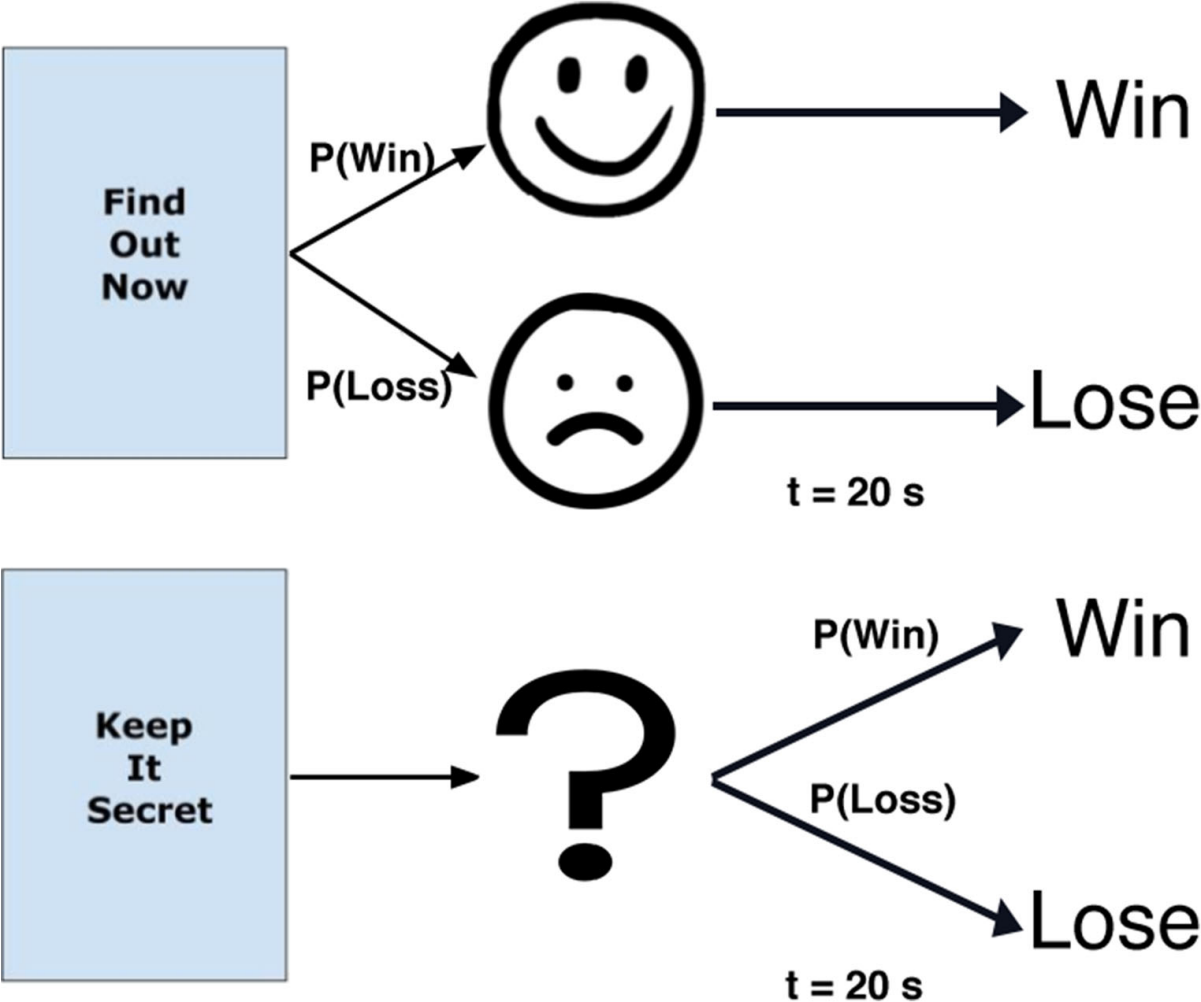
Experimental design of the secrets task used in the current experiment. By choosing Find Out Now participants immediately find out the result of the delayed outcome (via informative cues), whereas choosing Keep It Secret provides no information (an ambiguous cue is displayed) about the delayed outcome. Probability of a win/loss is varied along with the specific reward values such that their expected values are constant across conditions

In the present work, we adopt an exploratory approach to understanding the structure of general information preference by investigating the extent to which task-exogenous measures (i.e., trait scores) relate to task-endogenous factors (i.e., outcome probability) of non-instrumental information seeking in a standard paradigm known as the *secrets* task. Our version of the secrets task presents participants with a choice to either receive advance information (a cue) about the outcome of a risky event or to delay that information until the delivery of the outcome itself (see Fig. 1). Choosing the former results in the immediate presentation of an informative cue, while choosing the latter results in the presentation of an ambiguous cue. Both choices however require the participant to wait for a fixed amount of time before delivery of the outcome, and neither choice affects the probability of the outcome itself.

Several researchers have examined how a variety of endogenous factors affect information-seeking (e.g., Kobayashi, Ravaioli, Baranès, Woodford, & Gottlieb, 2019; van Lieshout, de Lange, & Cools, 2020; van Lieshout, Traast, de Lange, & Cools, 2021). We chose to examine outcome probability because previous research has found mixed effects of this task-endogenous factor on non-instrumental information preferences. Iigaya et al., (2020) investigated the effect of cue-outcome delay and outcome probability on information seeking behaviour and found that cue-outcome delay, but not outcome probability, predicted information preferences. In contrast, using a more complex secrets task where the presentation of cues was itself probabilistic (i.e., the predictive cues had some probability of being presented or not), Charpentier, Bromberg-Martin, and Sharot (2018) found that as the probability of winning $1 increased so did the tendency to seek information. Similarly, increasing the probability of negative rewards (losing $1) increased people’s tendency to avoid information. In their task the outcome magnitudes were fixed at constant values (obtaining or losing either $1 or $0), therefore the expected values tracked outcome probability.

Our experiment builds on this work in two major directions. First, we explore the relationship between task-endogenous variation in the secrets task with task-exogenous self-report measures of information-seeking behaviour. We present a total of five self-report scales: the Big Five Inventory-2 (Soto & John, 2017), Information Preferences Scale (Ho, Hagmann, & Loewenstein, 2021), Intolerance of Uncertainty-12 Scale (Carleton, Norton, & Asmundson, 2007), Five-Dimensional Curiosity Scale (Kashdan et al., 2018), and the Obsessive Compulsive Inventory (Foa et al., 2002). These scales were chosen for their prior application to other information-seeking tasks (e.g., Bennett et al.,, 2021; Jach et al.,, 2021; Jach & Smillie, 2021) as well as their intended purpose to measure trait-level information preferences (Ho et al., 2021). Second, we induce task-endogenous variation by using a secrets task that systematically varies outcome probability while holding expected value constant. This allows us to test whether non-instrumental information preferences are sensitive to outcome probability per se (and not merely expected value). Following prior results (Jach et al., 2021; Jach & Smillie, 2021), we expected to observe that information preference would track outcome probability and that the strongest associations would be between information preference and self-reported levels of uncertainty intolerance, curiosity, and openness. Finding this pattern of results would lend weight to the idea of a unitary construct of information preference that can be revealed via both behavioural and self-report measures; failing to observe this link would raise questions about the extent to which information preference should be considered as a singular, measurable entity.

## Experiment

### Methods

#### Participants

Participants were recruited via the University of New South Wales SONA recruitment platform. We sought to obtain a sample size in line with studies that have examined similar relationships (i.e., Bennett et al.,, 2021; Jach et al.,, 2021). Consequently, we analysed data from a sample of 279 participants (*M*_*age*_ = 19.2 years; 190 females, 88 males, and 1 other), after excluding data from 22 participants who failed the secrets task instructions check more than three times and 26 other participants who wrongly answered more than one out of five attention checks distributed within the self-report questionnaires.

#### Materials and procedure

After indicating their consent to participate, participants were presented with a series of written instructions for the experimental task. The instructions emphasised that the choice for advance information would not influence the outcome of the gamble. After reading the instructions participants completed a short three-item multiple choice quiz to ensure they understood the instructions. Submitting any incorrect answers would send participants back to the start of the instructions, following which they had to complete the same quiz again. Participants were allowed an unlimited number of attempts at the quiz, although as indicated earlier we only analysed data from participants who failed the quiz no more than three times.

Each trial of the experimental task presented participants with a gamble comprising different combinations of probabilities and outcomes designed to have a fixed expected value of 500 (gain condition) or -500 (loss condition). The probability of an outcome could take one of five levels: .01, .25, .50, .75, or .99, with the probability of the remaining outcome being its complement. In both conditions, one of the outcome magnitudes was fixed at 0 points, with the remaining outcome magnitude being determined by the probability distribution to maintain the fixed expected value (e.g., a gains condition trial may present a .25 probability of winning 2000 points and .75 probability of winning 0 points, resulting in an expected value of 500 points). The full series of different gambles are presented in Table 1.

**Table 1:** Distribution of gamble properties for gain and loss conditions

Pr(Gain or Loss)	Reward Magnitude
.01	50 000
.25	2000
.50	1000
.75	667
.99	505

Rewards are positive for the gain condition and negative for the loss condition. The complementary outcome is always 0. The expected value for each gamble is constant at 500 (gains) and − 500 (losses)

Along with the gamble, participants were also presented with two options relevant to receiving advance information on the gamble: they could either *Find Out Now* (FON) or *Keep It Secret* (KIS). Choosing FON resulted in the immediate presentation of an informative cue (either a smiley or sad face) that indicated the outcome of the gamble, while choosing KIS presented an ambiguous cue (a question mark). A smiley face was used as the cue for the more positive outcome (winning more than 0 points in the gains condition and receiving exactly 0 points in the loss condition) and a sad face was used for the more negative outcome (losing more than 0 points in the loss condition and receiving exactly 0 points in the gains condition; see Fig. 1). The number of points won or lost on that trial was presented to participants 20 seconds after the cue regardless of their prior choice for information. This specific cue-outcome delay was chosen for its reliability in reproducing information-seeking behaviour (Iigaya et al.,, 2020; Iigaya, Story, Kurth-Nelson, Dolan, & Dayan, 2016; Liew, Embrey, Navarro, & Newell, 2022; Zhu, Xiang, & Ludvig, 2017). The total number of points accumulated was also consistently presented to participants on the top right corner of the experimental display.

Participants were each presented with a total of 50 trials for each condition (i.e., win and loss), comprised 10 trials of each of the five outcome probability levels. The order of trials was randomised for each participant. In the gains condition, participants started the session with 0 points; in the loss condition, participants started the session with 50,000 points. Each participant was exposed to both gain and loss conditions as separate blocks, with the block order randomised between participants.^1^ This resulted in participants each observing 100 trials (50 trials per block).

Upon completion of the secrets task, participants were directed to a series of five self-report scales measuring constructs relevant to information-seeking. We presented the following scales with the presentation order randomised between and within scales:

- The Big Five Inventory-2 (Soto & John, 2017), a 60-item scale measuring the Big Five personality traits of openness (B5o), conscientiousness (B5c), extraversion (B5e), agreeableness (B5a), and negative emotionality (B5n). Each item is presented as a statement (e.g., “I am someone who is relaxed, handles stress well”) to which responses are provided on a 5-point Likert scale (ranging from “Disagree strongly” to “Agree strongly”).
- The Information Preferences Scale (IPS; Ho et al., 2021), a 13-item scale measuring the propensity for obtaining information using various hypothetical scenarios (e.g., “Ten years ago, you had the opportunity to invest in two retirement funds: Fund A and Fund B. For the past 10 years, you have invested all your retirement savings in Fund A. Do you want to know the balance you would have if you had invested in Fund B instead?”). Responses are collected on a 4-point scale (from “Definitely don’t want to know” to “Definitely want to know”). Notably, these items tend to probe for instrumental information—-that is, in each of the hypothetical scenarios the information can be used to guide future action relevant to the situation.
- The Intolerance of Uncertainty-12 Scale (Carleton et al., 2007), a 12-item scale measuring the acceptability of uncertain events. The 12 items are separated into two factors: those related to the anticipation (or dread) of future events (e.g., “One should always look ahead so as to avoid surprises.”), known as the Prospective Anxiety (IUSp) subscale; and those related to the inhibition of action, known as the Inhibitory Anxiety (IUSi) subscale (e.g., “When it’s time to act, uncertainty paralyses me.”). Responses are provided on a 5-point scale ranging from “not at all characteristic of me” to “entirely characteristic of me”.
- The Five-Dimensional Curiosity Scale (Kashdan et al., 2018), a 25-item scale measuring five separate curiosity factors: deprivation sensitivity (CURds; e.g., “It frustrates me not having all the information I need.”), joyous exploration (CURje; e.g., “I find it fascinating to learn new information.”), stress tolerance (CURst; e.g., “The smallest doubt can stop me from seeking out new experiences.”), social curiosity (CURsc; e.g., “I like to learn about the habits of others.”), and thrill seeking (CURts; e.g., “I prefer friends who are excitingly unpredictable.”).
- The Obsessive Compulsive Inventory (OCI; Foa et al., 2002), a 18-item scale assessing symptoms of obsessive compulsive disorder. Each item is presented as a short scenario (e.g., “I repeatedly check doors, windows, drawers, etc.”), and participants are asked how distressing they find each scenario, with responses collected on a 5-point scale ranging from “Not at all” to “Extremely”.

### Results

**Fig. 2:**
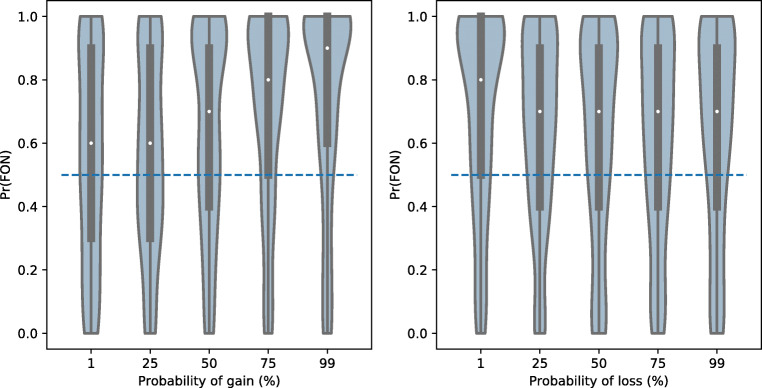
Violin plots indicating the distribution of Find Out Now (FON) responses with increasing probability of the non-zero outcome. Densities of FON responses are indicated by the blue regions for each level of outcome probability, with white markers indicating the median. The blue dashed line indicates the point of information indifference (i.e., Pr(FON) = .5)

Figure 2 presents the distribution of FON choices across increasing probability levels for both the gain (left) and loss (right) conditions. Preferences for information generally increased with the probability of a gain, and generally decreased, although not as systematically, with the probability of a loss.

Generalised linear mixed models (GLMM), using participants as the grouping factor, were used to analyse the effect of outcome probability and valence (i.e., gain or loss) on FON choice proportion. We compared a series of statistical models of increasing complexity, starting from: 1) a baseline model containing only random intercepts with no predictors, 2) a random intercept model with probability as a predictor, 3) a random slope model using only probability as a predictor, 4) a random slope model with all task-endogenous predictors (probability and valence), and finally 5) a random slope model with all task-endogenous and all task-exogenous predictors (i.e., scores on the self-report questionnaires). Model 4 emerges as our preferred model on the basis of having the lowest BIC value as well as it being significantly better-fitting than simpler models (Table 2). Parameter estimates of Model 4 are presented in Table 3, from which we can see that there are significant fixed effects of both predictors and their interaction. Specifically, not only do we find an increase in preference for information with increasing probability of winning, we also observe a lower mean FON response for gains compared to losses. The significant interaction between outcome probability and valence indicates what is clearly presented in Fig. 2: that increases in outcome probability drives information seeking higher in gains but lower in losses.

**Table 2:** GLMM model comparison

#	Linear Model	Predictors	BIC	*χ*^2^	df	*p*
1.	Random intercept	−	31633	−	−	−
2.	Random intercept	Probability	31561	81.79	1	< .001
3.	Random slope	Probability	31134	447.13	2	< .001
4.	Random slope	All Endogenous^∗^	30423	762.83	5	< .001
5.	Random slope	All Endogenous and Exogenous^+^	30544	22.41	14	.07

^*^ Task-endogenous: probability, valence

^+^ Task-exogenous: B5o, B5c, B5e, B5a, B5n, IPS, IUSp, IUSi, CURds, CURje, CURst, CURsc, CURts, OCI

**Table 3:** Estimates of fixed effects of Model 4

Parameter	Estimate	SE	*z*	*p*
Intercept	0.912	0.096	9.51	< .001
Probability	− 0.003	0.001	− 3.36	< .001
Valence	− 0.709	0.075	− 9.49	< .001
Interaction	0.015	0.001	17.98	< .001

**Fig. 3:**
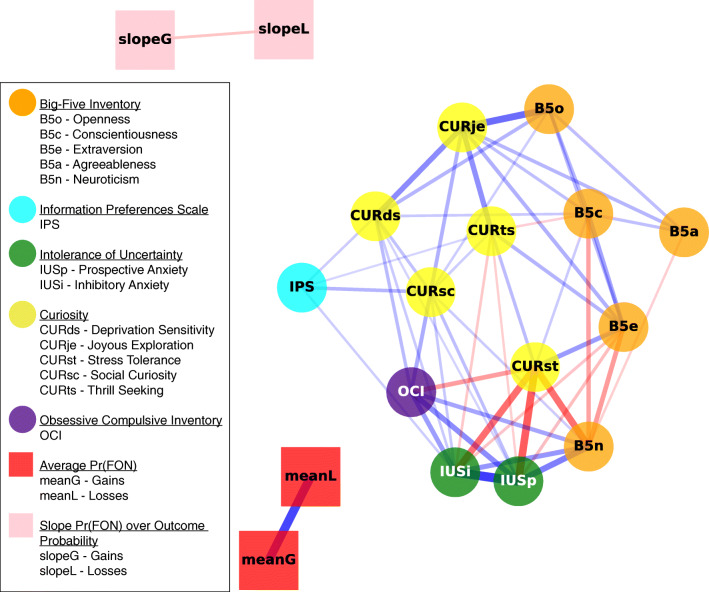
Force-directed graph showing correlations between measures related to task-endogenous (square nodes) and task-exogenous (circular nodes) variables. Measures with greater correlation tend to be located closer together. Strength of correlations is indicated by the thickness of blue (positive) and red (negative) edges (connecting lines) respectively. Only correlations with more than moderate evidence (i.e., *BF*_01_ < .3) are displayed. MeanG and meanL represent the mean information preference in gains and losses respectively. SlopeG and slopeL represent the slope of information preference (over increasing probability of a gain/loss) for gains and losses respectively. FON refers to Find Out Now responses

Correlations between every task-exogenous self-report variable as well as task-endogenous-relevant information-seeking responses are displayed in Fig. 3.^2^ Statistical inferences on correlations are made with Bayes factors (*BF*_01_) with interpretations guided by Lee and Wagenmakers (2014). Several features of Fig. 3 are noteworthy.^3^ First, it is clear that while the task-exogenous measures are mostly co-located, both within and between different measures, the task-endogenous nodes which capture choices in the secrets task lie apart from this network. In other words, there is little to suggest a relationship between information preference in the secrets task and the self-report measures. Second, we unsurprisingly see that preference for information (FON) in the loss and gain version of the secrets task are strongly positively correlated (*r* = .73), whereas the slopes of FON across increasing probability levels are negatively correlated (*r* = −.23) between gains and losses (reflecting the overall pattern of preferences seen in Fig. 2 within individuals). Third, we see sensible and interpretable relationships between task-exogenous measures, such as the negative relationship between the stress tolerance curiosity subscale with intolerance of uncertainty and negative emotionality (CURst with IUSi, IUSp, and B5n; *rs* < −.56). Joyous exploration also correlated strongly with openness (CURje and B5o, *r* = .57).

## Discussion

We examined whether information preference should be considered a unitary measurable construct. The results of our experiment comparing behavioural and self-report measures of the tendency to seek out information strongly suggest that information preference is non-unitary. Our experimental paradigm - the secrets task - reliably produced the expected systematic variation in information-seeking behaviour across different outcome probabilities and valences. However, this individual-level variation in task performance was at best only weakly associated with trait-level measures of information preference.

This absence of an association was not (necessarily) due to poor selection of task-endogenous variables. Correlations observed between the self-report measures are close reproductions of similar results found in prior experiments. For instance, the relatively strong positive correlation between openness and joyous exploration as well as that between intolerance of uncertainty and deprivation sensitivity was also observed in Jach and Smillie (2021) and Jach et al., (2021). The strong positive correlations between obsessive-compulsion, intolerance of uncertainty, and negative emotionality were also found in those prior studies, as well as in Bennett et al., (2021). While the task-exogenous variables generally correlated strongly with each other, the almost nonexistent association between task-endogenous and task-exogenous variables adds to an increasingly complex picture of information preference. People’s general levels of curiosity, negative emotionality, and self-reported information preferences appear independent from task-specific non-instrumental information-seeking behaviour.

This pattern of results may seem problematic at first glance. For instance, it raises the question of which other factors might account for such individual variation. However, this observation is far from uncommon—it aligns with similar studies noting significant individual differences with little to no association with trait measures (Bennett, Bode, Brydevall, Warren, & Murawski, 2016; Bennett et al., 2021; Brydevall, Bennett, Murawski, & Bode, 2018; Kobayashi et al., 2019). Further, the similarity in our findings (and sample sizes) with prior studies employing different non-instrumental information-seeking tasks (e.g., Bennett et al., 2021; Jach et al., 2021; Jach & Smillie, 2021) provides converging evidence which suggests task-endogenous and task-exogenous factors are independent. More generally, such non-unitary observations of apparently singular entities have also been observed in other psychological constructs such as risk preference (Frey, Pedroni, Mata, Rieskamp, & Hertwig, 2017). It is possible that the individual differences observed here are the result of idiosyncratic approaches to the task—that is, the variation across individuals is fundamentally “noise” that cannot be captured by any of the current trait-level measures. Indeed, it is an open question whether the variation could be captured by other candidate task-exogenous factors, such as Need for Cognition (Holanda Coelho, Hanel, & Wolf, 2020) or measures of impulsivity (e.g., BIS; Patton, Stanford, & Barratt, 1995), or whether the task is simply not a good correlate of more general traits of information-preference and decision-making.

An alternative perspective is to consider the “noise” in behavioural responding as variability that should be focused on and explained through the lens of participants’ hypotheses about the relevant aspects of a given task or situation. The similarity with investigations of risk-preference is salutary here: more experiential (trial-by-trial) measures of risk and information-tendencies may show weak correlations with self-report measures precisely because the former invite participants to seek out explanations for experimental features such as the potential for repeating patterns, dependencies in outcomes, or hypotheses about what the experiment is testing that are absent in task-exogenous assessments (Szollosi & Newell, 2020). Whether one or other type of test is thus a ‘better’ measure of the construct of interest depends perhaps on whether one is attempting to measure constructed or static preferences (Frey et al., 2017; Kobayashi et al., 2019).

Interestingly, stronger correlations have been observed between traits and behaviour associations when an explicit cost is incurred by obtaining information (Jach et al., 2021). Bennett et al., (2021) found that when modelling costly information-seeking behaviour, task-endogenous information preferences were positively correlated with task-exogenous measures of obsessive-compulsion and negative emotionality. In addition, they found that willingness to obtain costless information was negatively correlated with willingness to obtain costly information—this was observed both within their dataset as well as data from Brydevall et al., (2018) and Bennett et al., (2016). An explanation for the diverging effects of costly and costless information is still unclear, although it may plausibly be the result of employing different decision processes for costly and costless information. For instance, within the framework of Jach et al., (2021)’s dual-pathway account of information-seeking, costly information may evoke further engagement with the safety pathway than when information is costless, resulting in stronger associations between safety-related traits and costly information (as was observed in their study). Conversely, costless information may involve more engagement with the exploration pathway than when information is costly, consequently increasing association between exploration-related traits and costless information. One could also think of the cost of information as a cue that constrains participants’ hypotheses about the relevant features of task thereby potentially reducing the variation in responding and increasing the likelihood of finding significant associations with trait measures. Further investigations of these ideas are warranted.

Turning briefly to an additional aspect of our results, our key behavioural finding showing information-seeking behaviour increases with the probability of the better outcome appears to reaffirm that this effect observed in Charpentier et al., (2018) is driven by changes in outcome probability. While we cannot rule out an effect of expected value on non-instrumental information seeking (since we did not vary expected value independently from outcome probability), the alignment of the observed differences in information seeking with prior literature (e.g., Charpentier et al., 2018; Embrey, Liew, Ghai, & Newell, 2021) suggest a dominance of the effect of probability over any possible effect of expected value. More generally, this is also supported by growing empirical evidence indicating the lack of an effect of the magnitude and expected value of reward in information seeking (Cabrero, Zhu, & Ludvig, 2019; van Lieshout, Vandenbroucke, Müller, Cools, & de Lange, 2018; Liew & Newell, 2021) .

Ultimately, our data suggest that it is insufficient to think of general information preference as a unitary latent construct that can be similarly captured by both task-endogenous and task-exogenous measures. Just as other major psychological constructs such as intelligence and risk preference have been identified with stable components alongside situation-specific measures (e.g., Frey et al., 2017; Spearman, 1904), it may also be more helpful to understand information preference as a multi-faceted construct with task-specific components that can be independent from broader trait-level factors.

## Open Practices Statement

Neither of the experiments reported in this article were formally preregistered. The data and the materials have been made available at this location: https://osf.io/mkeb9x.

### Electronic supplementary material

Below is the link to the electronic supplementary material.

(PDF 91.5 KB)

## Footnotes

1 Recent literature has indicated that the randomisation of presentation order within participants can obscure relationships between task-endogenous and task-exogenous measures (Goodhew & Edwards, 2019). Given that previous experiments similar to the present study have demonstrated observable effects using randomised presentation orders (Bennett et al., 2021; Jach et al., 2021), we saw no reason to use a fixed order of block presentation here.

2 Specific correlation values are presented in Table 1 of the Appendix.

3 Specific *B**F*_01_ values are presented in Table 2 of the Appendix.
