# Supplementary material for: The non-unitary nature of information preference
Source: Psychon Bull Rev. 2023 Apr 19;30(5):1966–74. doi: 10.3758/s13423-022-02243-5 (PMC10716071; doi:10.3758/s13423-022-02243-5)
Supplement: Supplementary file 1 — (PDF 91.5 KB) [file 13423_2022_2243_MOESM1_ESM.pdf]

Appendix

Table 1: Pearson's Correlations Between Each Task-Exogenous and Task-Endogenous Measure

Measure	meanG	meanL	slopeG	slopeL	B5o	B5c	B5e	B5a	B5n	IPS	IUSp	IUSi	CURds	CURje	CURst	CURsc	CURts	OCI
meanG	–																	
meanL	.73***	–																
slopeG	−.00	.02	–															
slopeL	.07	.10	−.23***	–														
B5o	−.03	−.05	−.00	.13	–													
B5c	−.02	−.08	.03	.03	.28***	–												
B5e	−.03	−.08	.03	.01	.27***	.35***	–											
B5a	−.01	−.06	−.01	.06	.26***	.25***	.16	–										
B5n	.07	.11	.04	.00	−.04	−.36***	−.39***	−.19**	–									
IPS	.07	.10	−.00	.07	.07	−.15	.07	.01	.02	–								
IUSp	.11	.10	.01	.02	−.13	−.16	−.26***	−.16	.50***	.12	–							
IUSi	.12	.11	.01	.01	−.16	−.02	−.23***	−.16	.41***	.19**	.76***	–						
CURds	.16	.12	.02	.11	.29***	.22**	.12	.00	.13	.20**	.18*	.21**	–					
CURje	.06	.06	.02	.02	.57***	.25***	.31***	.29***	−.07	.11	−.03	−.06	.45***	–				
CURst	−.03	−.01	−.05	.01	.16	.21**	.39***	.08	−.56***	−.02	−.63***	−.56***	−.11	.11	–			
CURsc	−.02	−.00	.04	−.01	.20**	−.10	.07	.03	.21**	.29***	.25***	.22**	.24***	.31***	−.15	–		
CURts	−.04	.01	−.02	.03	.16	−.19**	.30***	−.02	−.09	.17*	−.19**	−.22**	.11	.43***	.26***	.20**	–	
OCI	.06	.07	.05	.08	.03	−.13	−.13	−.13	.35***	.13	.45***	.39***	.27***	.10	−.38***	.32***	.10	–

* $BF_{01} < .3$, ** $BF_{01} < .1$, *** $BF_{01} < .01$

Table 2: Bayes Factors (BF_{01}) Corresponding to Correlations Between Each Task-Exogenous and Task-Endogenous Measure

Measure	meanG	meanL	slopeG	slopeL	B5o	B5c	B5e	B5a	B5n	IPS	IUSp	IUSi	CURds	CURje	CURst	CURsc	CURts	OCI
meanG	–																	
meanL	$< .01$	–																
slopeG	13.32	12.56	–															
slopeL	6.44	3.27	$< .01$	–														
B5o	11.52	9.25	13.32	1.07	–													
B5c	12.74	5.81	11.56	12.03	$< .01$	–												
B5e	12.21	5.94	11.99	12.94	$< .01$	$< .01$	–											
B5a	13.20	8.18	13.19	7.61	$< .01$	$< .01$	.38	–										
B5n	6.57	2.84	10.77	13.34	10.57	$< .01$	$< .01$	.07	–									
IPS	7.14	3.69	13.31	7.18	7.18	.64	6.96	13.19	12.47	–								
IUSp	2.33	3.37	13.15	12.73	1.42	.35	$< .01$	.43	$< .01$	1.91	–							
IUSi	1.81	2.51	13.29	13.23	.35	12.53	$< .01$	.42	$< .01$	.07	$< .01$	–						
CURds	.40	1.70	12.50	2.17	$< .01$	.02	1.71	13.34	1.52	.04	.16	.03	–					
CURje	7.61	8.52	12.88	12.37	$< .01$	$< .01$	$< .01$	$< .01$	6.70	2.50	11.57	8.59	$< .01$	–				
CURst	11.79	13.10	9.90	13.17	.40	.03	$< .01$	5.27	$< .01$	12.37	$< .01$	$< .01$	2.18	2.49	–			
CURsc	12.26	13.34	11.10	13.01	.06	3.48	7.02	11.58	.03	$< .01$	$< .01$	.01	$< .01$	$< .01$	.55	–		
CURts	10.26	13.27	12.54	12.21	.43	.07	$< .01$	12.82	3.91	.23	.09	.02	2.48	$< .01$	$< .01$	.05	–	
OCI	7.60	6.47	10.04	6.04	11.97	1.38	1.20	1.17	$< .01$	1.14	$< .01$	$< .01$	$< .01$	3.05	$< .01$	$< .01$	3.77	–

* Note that higher BF_{01} values (specifically $BF_{01} > 1$) indicate greater evidence for the null hypothesis (i.e., no correlation) while lower BF_{01} values ($BF_{01} < 1$) indicate greater evidence for the alternative hypothesis (correlation observed).
